# Children’s continued usage intention in virtual reality-based pottery courses: a study on the collaborative mechanism of cognitive engagement and affective arousal

**DOI:** 10.3389/fpsyg.2026.1683244

**Published:** 2026-06-10

**Authors:** Chaoran Tong, Lei Que, Hongyan Guan, Xu Zhu

**Affiliations:** 1 School of Fine Arts and Design, Shaoguan University, Shaoguan, Guangdong, China; 2 College of Arts, Guilin University of Technology, Guilin, Guangxi, China; 3 Faculty of Creative Technology and Heritage, Universiti Malaysia Kelantan, Kota Bharu, Kelantan, Malaysia

**Keywords:** affective arousal, children’s education, cognitive engagement, continued usage intention, flow experience, satisfaction, stimulus-organism-response framework, virtual reality

## Abstract

**Introduction:**

Sustaining children’s engagement in immersive learning environments is a central challenge in educational psychology. Virtual reality (VR) offers multisensory and interactive experiences that can foster creativity and skill development. However, the psychological mechanisms that drive sustained participation remain underexplored. This study applied the stimulus–organism–response (S–O–R) framework to examine how cognitive engagement (e.g., attention guidance and cognitive load control) and affective arousal (e.g., valence design and arousal intensity) influence children’s flow experience, satisfaction, continued engagement, and perceived learning achievement in VR-based pottery education.

**Methods:**

A total of 220 elementary school students (aged 7–12 years) from four Chinese cities participated in a structured VR pottery course. Following the activity, participants completed validated questionnaires measuring the targeted constructs. Data were analyzed using partial least squares structural equation modeling, with bootstrapping employed to assess path significance and model fit indices to evaluate the overall adequacy.

**Results:**

Cognitive engagement had a stronger positive impact on flow and satisfaction than affective arousal. Flow was a stronger predictor of continued engagement than satisfaction. Continued engagement significantly predicted perceived learning achievement. The structural model demonstrated acceptable fit (standardized root mean square residual = 0.062), with substantial variance explained in key outcome variables.

**Discussion:**

These findings highlight the dominant role of cognitive engagement in fostering sustained participation in VR-based creative learning, with affective arousal providing supplementary enhancement. The results extend the stimulus–organism–response framework to children’s art education, offering theoretical insights into cognitive-affective mechanisms and practical guidance for designing psychologically optimized VR learning environments.

## 1 Introduction

Ceramic art education, as a vital carrier of human cultural heritage, has long held a unique position in children’s art education (UNESCO, 2023). Currently, ceramic creation through practices such as three-dimensional shaping and glazing plays a key role in developing children’s hand–eye coordination, non-verbal expression, and intercultural aesthetic literacy (Brodzeller et al., 2018). Globally, pottery courses have become a core component of elementary-level art education (MEXT, 2017; UNESCO, 2023). However, traditional instructional models face three major practical challenges. First, pottery requires high material consumption, with each student typically using 0.8–1.2 kg of clay per session and firing failure rates exceeding 30% (UNESCO, 2022). Second, kiln operation carries a risk of burns. According to the U.S. Consumer Product Safety Commission, ceramic classroom-related burns comprise 19% of injuries in elementary school craft classes (CPSC, 2019). Third, complex modeling is constrained by physical space and tool precision (Green, 2014). These limitations increase instructional costs and hinder curriculum accessibility and creative depth.

Compared to other art forms, pottery making is characterized by its highly embodied nature and multisensory integration. Pottery making requires children to engage in fine hand–eye coordination and spatial imagination and elicits strong emotional responses through tactile feedback, material transformation, and witnessing their creation take shape. This interweaving of cognition and emotion within a hands-on activity makes pottery an ideal vehicle for examining children’s cognitive–affective interactions in immersive environments. Virtual reality (VR) technology is characterized by multidimensional features such as immersion, multimodal interactivity, and dynamic presence (Martin et al., 2021; Wei et al., 2025). These characteristics have demonstrated substantial potential across diverse fields, from healthcare to architecture, with clear advantages in education (Fusaro et al., 2025). Its immersive qualities, delivered through 110° field-of-view headsets and spatial audio, significantly enhance learner attention and extend children’s focus time by up to 2.3-fold in virtual environments compared with traditional classrooms (Cummings and Bailenson, 2016; Makransky et al., 2017; Tarng et al., 2022). Its interactivity further enables precise motion training through gesture recognition and haptic feedback, achieving operational accuracy within ±0.5 mm (Trinitatova and Tsetserukou, 2019; Weichert et al., 2013). Furthermore, its imaginative power allows learners to bypass physical constraints, such as observing molecular motion in chemistry or “traveling through” tectonic structures in geography (Martin et al., 2021). Empirical studies have shown that effectively integrating VR into diverse educational contexts can enhance learning immersion and knowledge retention.

VR offers an interactive, digitized training solution for traditional craft education by simulating real-world ceramic creation, thereby improving skill acquisition efficiency (Luo et al., 2025). Through high-precision 3D modeling and physics engines, virtual clay can be reused infinitely, reducing material loss to 0%, while kiln temperatures can be precisely controlled within ±1 °C via digital interfaces, effectively eliminating burn risks (Dashti et al., 2022). Moreover, VR environments support a level of creative freedom that is rarely achieved in traditional classrooms. For instance, children can use “gravity elimination” tools to create levitating pottery or employ “topological deformation” functions to adjust real-time clay thickness to as little as 0.1 mm, thereby enabling the mastery of advanced shaping techniques (Kim, 2023). However, such technological advantages do not necessarily translate into high user retention. A user study on the game “Pottery VR” found that although 63.7% of participants spent more than 5 min in their initial session, only 22.6% reported “frequent” repeat usage and 36.3% “rarely” used it again, suggesting relatively low user engagement. Prior research indicates that technology acceptance is shaped by multidimensional psychological drivers (Qiu et al., 2024). However, current VR pottery course designs tend to overemphasize performance optimization (Hui et al., 2022) while addressing the underlying psychological foundations of children’s learning behaviors, particularly to the alignment of cognitive load thresholds and the calibration of affective arousal levels with individual needs (Nasri, 2025). This gap calls for a systematic investigation.

The psychological mechanisms of learning behaviors can be conceptualized into two core dimensions: cognitive engagement and affective arousal (Moreno and Mayer, 2007). The cognitive engagement dimension focuses on knowledge acquisition and skill development, with attention guidance and cognitive load control serving as key mechanisms (Parong and Mayer, 2018). The affective arousal dimension emphasizes emotional experience and motivational activation, encompassing two central indicators: valence and arousal level (Mehrabian and Russell, 1974). The quality of the learning experience depends on the synergy between cognitive and affective responses (Mirvis, 1991). When these mechanisms are effectively aligned, learners are more likely to enter a state of flow, characterized by deep concentration, temporal distortion, and reduced self-awareness (Chou et al., 2023). Concurrently, they may experience satisfaction, which stems from goal achievement and manifests as a sense of competence and enhanced self-efficacy (Pekrun et al., 2007). Conversely, insufficient attention guidance or excessive cognitive load can lead to anxiety and frustration (Makransky et al., 2019a,b), while distorted valence or overstimulation may scatter attentional resources (Özgür and Altun, 2021). Therefore, the quality of learning experience is not a linear summation of dimensions but rather requires a dynamic balance between cognitive and affective pathways (Forsblom et al., 2022). Existing research has shown that the synergy between cognition and emotion is essential for optimizing VR-based learning experiences (Plass and Kaplan, 2016). However, VR pottery education studies have two key limitations. First, cognitive-focused research often oversimplifies the moderating effects of affective variables, neglecting how distorted valence can trigger behavioral avoidance (Schneider et al., 2022). Second, affective-focused research typically lacks longitudinal tracking of arousal overload, hindering explanations of the diminishing marginal effects of sensory stimulation (Vatsal et al., 2024). This disconnect has led to “technology-centered” design pitfalls, such as overreliance on particle effects to enhance arousal, which may ironically reduce learning motivation due to attentional dispersion (Park et al., 2014).

The stimulus–organism–response (S–O–R) framework, proposed by environmental psychologists Mehrabian and Russell (1974), provides a systematic paradigm for explaining how external stimuli drive behavioral intention through psychological states. Although alternative frameworks, such as self-determination theory or control-value theory of achievement emotions, offer valuable insights into learning motivation, the S–O–R framework is particularly suitable for this study. Specifically, it provides a systematic paradigm for deconstructing how externally designed stimuli (S) in a VR environment directly influence internal psychological states (O), which, in turn, drive behavioral outcomes (R). This clear causal chain is specifically suitable for analyzing technology-mediated learning environments in which specific design features are manipulated.

Furthermore, the S–O–R framework allows us to deconstruct how the stimuli of a designed VR environment influence children’s internal organismic states, leading to learning responses. Pottery making is a uniquely meaningful context for this investigation as it is fundamentally an embodied, sensorymotor activity. Its traditional pedagogical value derives from direct tactile engagement with clay, such as feeling its resistance, texture, and responsiveness to pressure. This presents a central challenge and opportunity for VR to replicate and reinterpret these material qualities. Our VR system compensates for missing tactile feedback through multimodal cues (e.g., visual deformation of virtual clay synchronized with hand movements, auditory scraping sounds, and haptic vibrations) to create a psychologically compelling analog to the physical experience. Therefore, this study does not assume that VR technology “replaces” real clay but rather explores how children emotionally and cognitively respond to this shift when VR technology is used to interpret pottery.

To clarify the core constructs under investigation, we define them within the context of this study. Flow experience refers to the state of deep, effortless involvement and distorted time perception during the VR pottery task (Csikszentmihalyi, 1990). Satisfaction is the *post hoc* affective appraisal of one’s overall experience and accomplishments. Attention guidance and cognitive load control constitute the cognitive engagement path, operationalized through system cues, such as visual highlights and task segmentation, to direct focus and manage mental effort (Parong and Mayer, 2018). The affective arousal path comprises valence and arousal (Mehrabian and Russell, 1974).

In VR education research, cognitive engagement (e.g., attention guidance and cognitive load control) and affective arousal (e.g., valence and arousal) have been extensively validated as key sources of stimulus (S), directly influencing the learning process by regulating cognitive load and attentional stability (Dubovi, 2022; Parong and Mayer, 2018). In this study, these constructs were assessed through parent-reported perceptions of the child’s experience during the VR pottery course. As organismic states (O), flow experience and satisfaction constitute core mediating mechanisms, and their synergy enhances motivational persistence (Csikszentmihalyi, 1990; Makransky and Lilleholt, 2018). Ultimately, these psychological states drive behavioral intention (R), which manifests as increased continuance intention and perceived learning achievement. Among these, behavioral persistence significantly promotes skill transfer and creativity development through cumulative training effects (Radianti et al., 2020; Sitzmann, 2011). In this model, flow experience and satisfaction are conceptualized as parallel mediating mechanisms. Flow represents a state of deep, process-oriented immersion during a task, whereas satisfaction reflects a more evaluative, outcome-based appraisal after one’s task experience. Although they are often related, prior research suggests that they can have independent effects on continued intention (Chiu and Chen, 2023; Makransky and Lilleholt, 2018). Exploring these factors in parallel enabled us to examine the distinct contributions of immersive engagement and *post hoc* evaluation in driving children’s willingness to continue using VR.

Therefore, this study proposes the following hypotheses:

- H1a: Attention guidance has a positive effect on flow experience.
- H1b: Attention guidance has a positive effect on satisfaction.
- H2a: Cognitive load control has a positive effect on flow experience.
- H2b: Cognitive load control has a positive effect on satisfaction.
- H3a: Valence design has a positive effect on flow experience.
- H3b: Valence design has a positive effect on satisfaction.
- H4a: Arousal design has a positive effect on flow experience.
- H4b: Arousal design has a positive effect on satisfaction.
- H5: Flow experience has a significant positive effect on children’s continued usage intention in VR-based pottery courses.
- H6: Satisfaction has a significant positive effect on children’s continued usage intention in VR pottery courses.
- H7: Behavioral intention has a significant positive effect on children’s perceived learning achievement in VR pottery courses.

**Figure 1:**
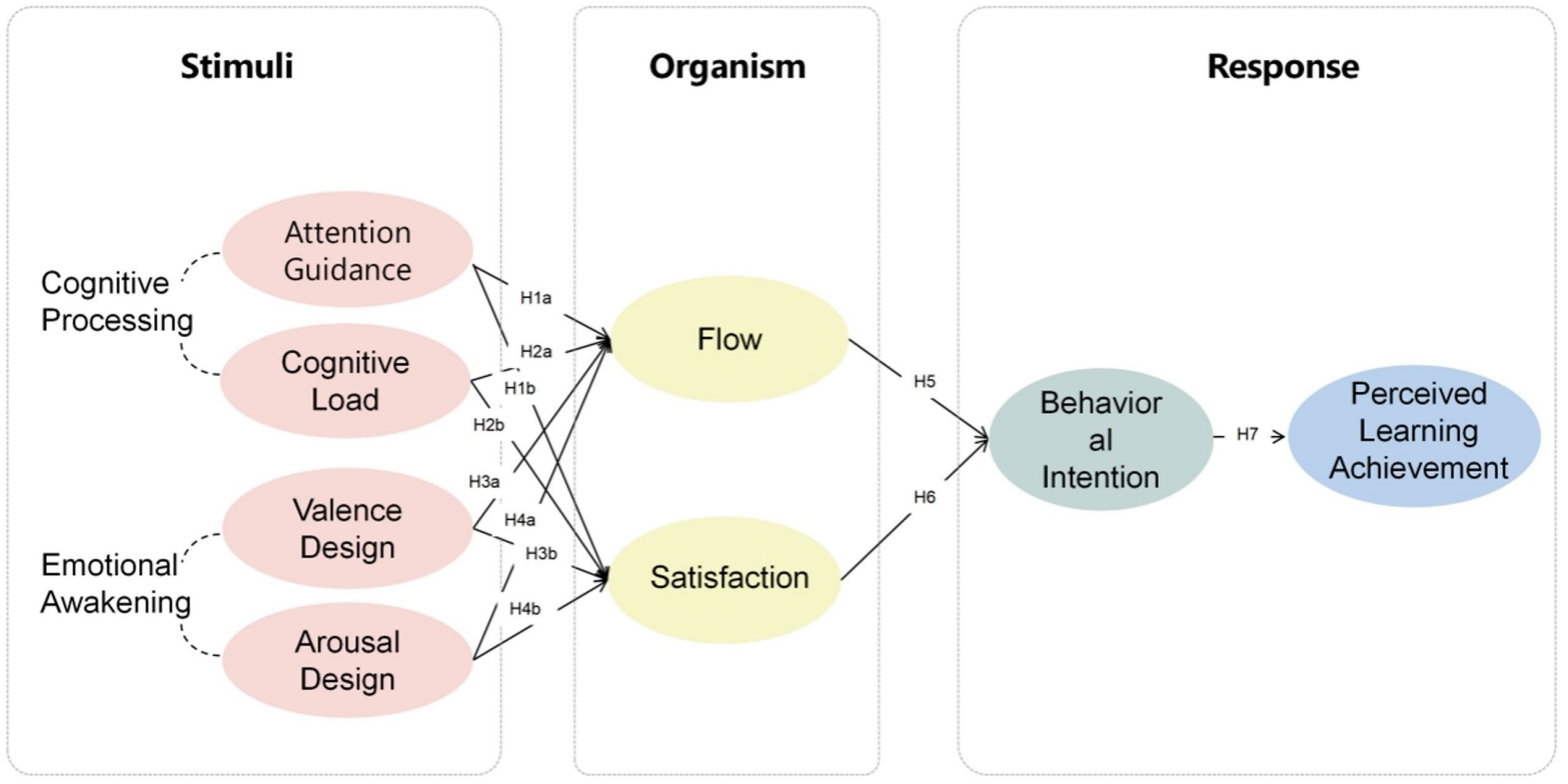
Proposed conceptual framework based on the stimulus–organism–response (S–O–R) model illustrating the relationships among cognitive engagement, affective arousal, flow experience, satisfaction, behavioral intention, and perceived learning achievement in VR-based pottery courses.

The proposed conceptual framework based on the above is illustrated in Figure 1.

## 2 Materials and methods

### 2.1 Experimental equipment and system design

**Figure 2:**
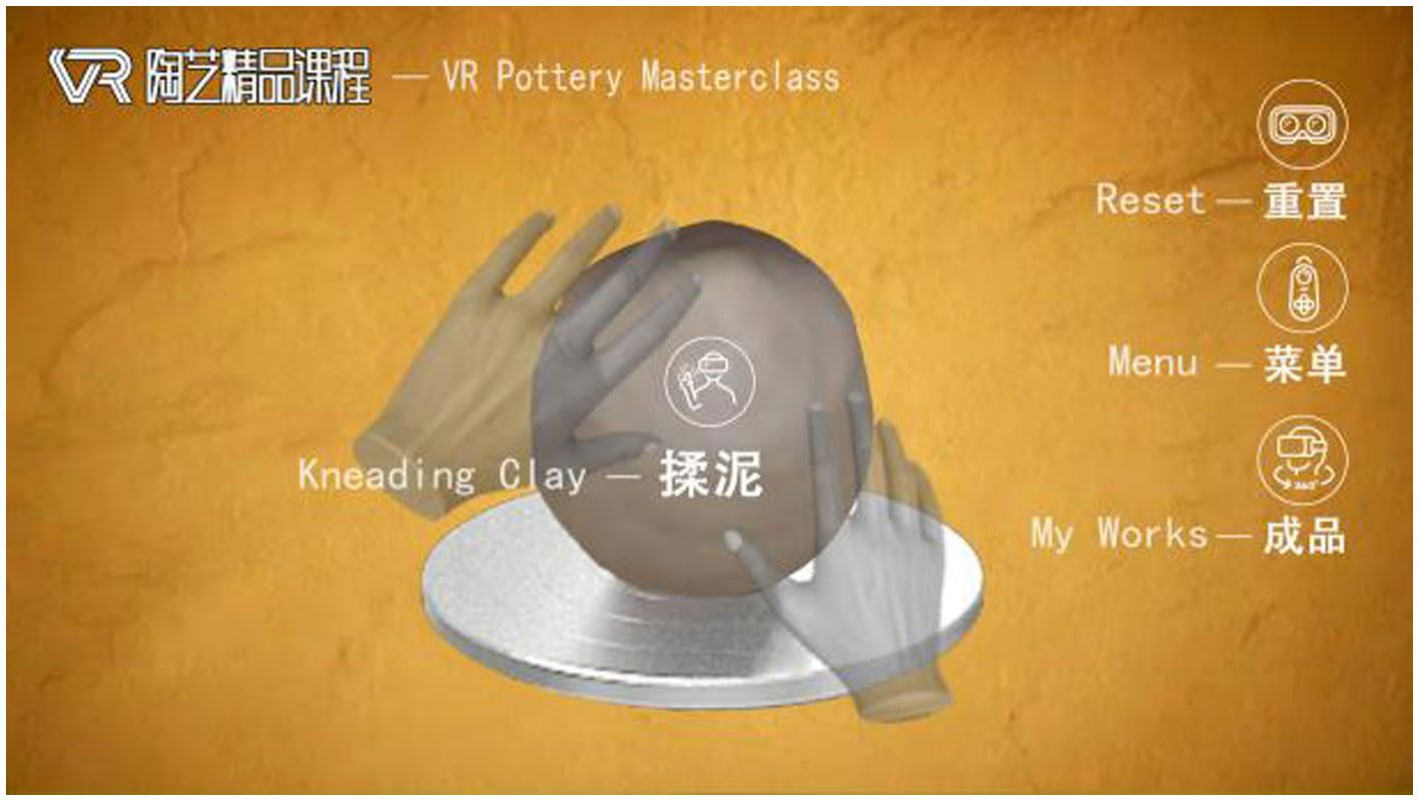
VR pottery interface showing the clay shaping process with hand interaction and operational guidance elements.

**Figure 3:**
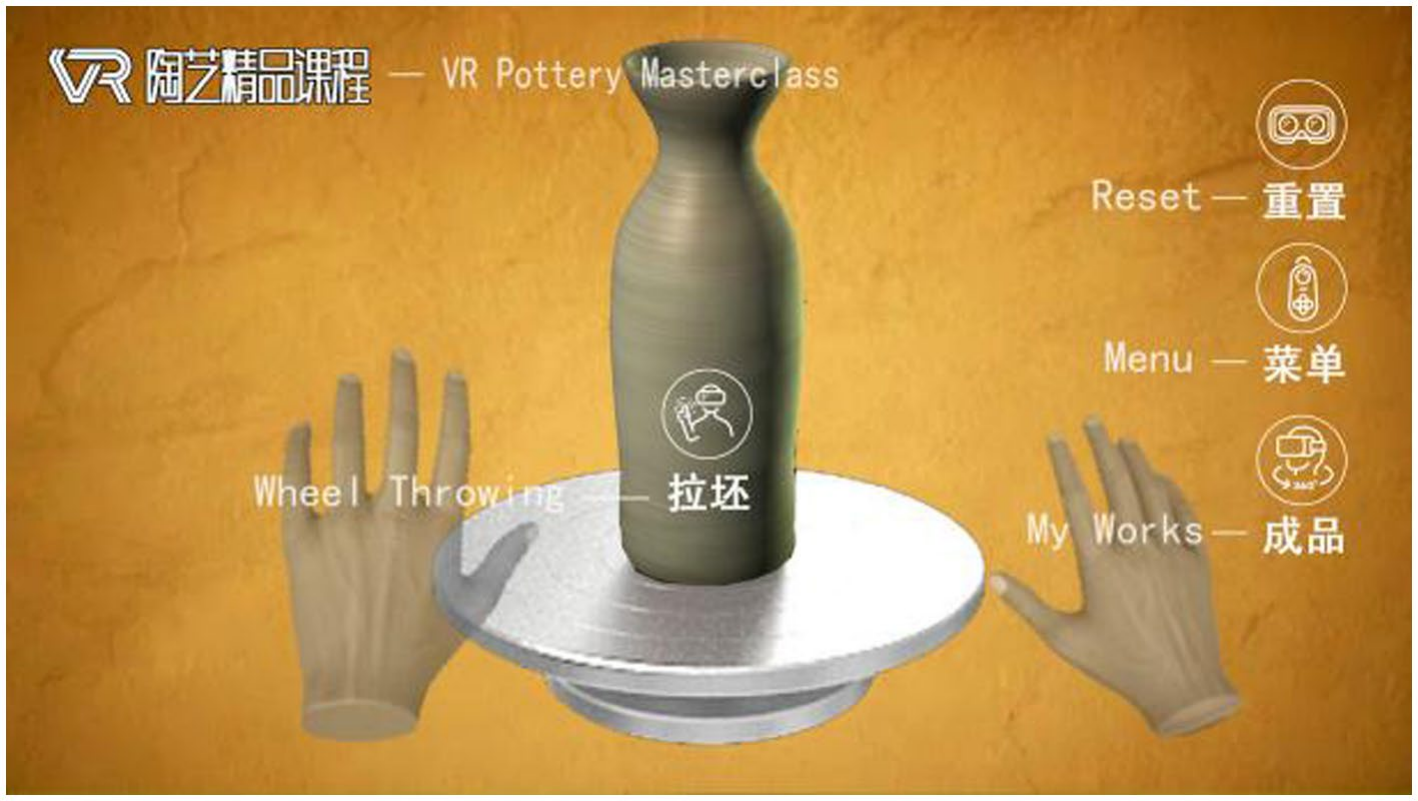
Main operational interface of the VR pottery system displaying wheel throwing interaction and menu options.

**Figure 4:**
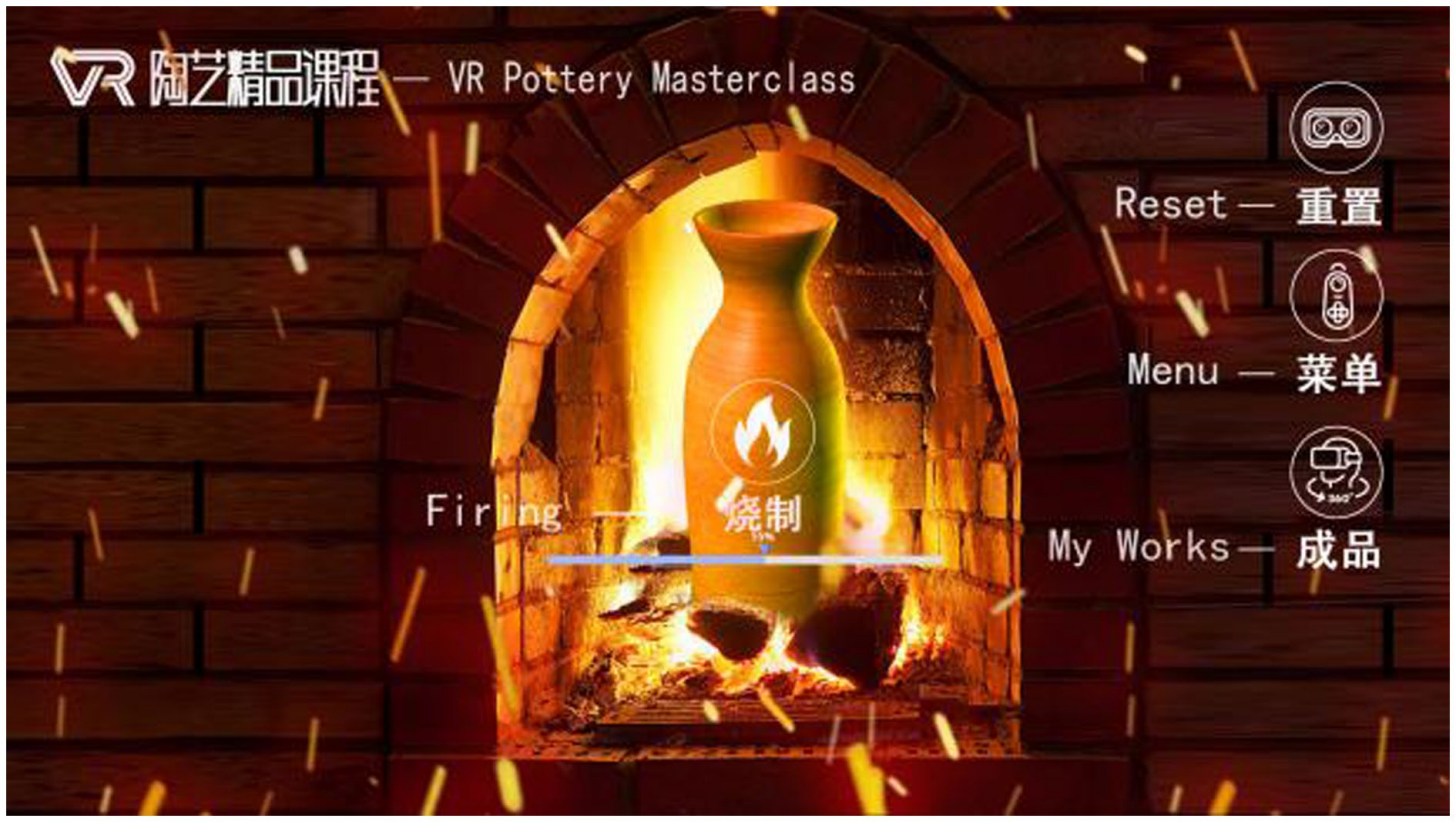
Virtual firing stage in the VR pottery course showing kiln effects and immersive environmental feedback.

**Figure 5:**
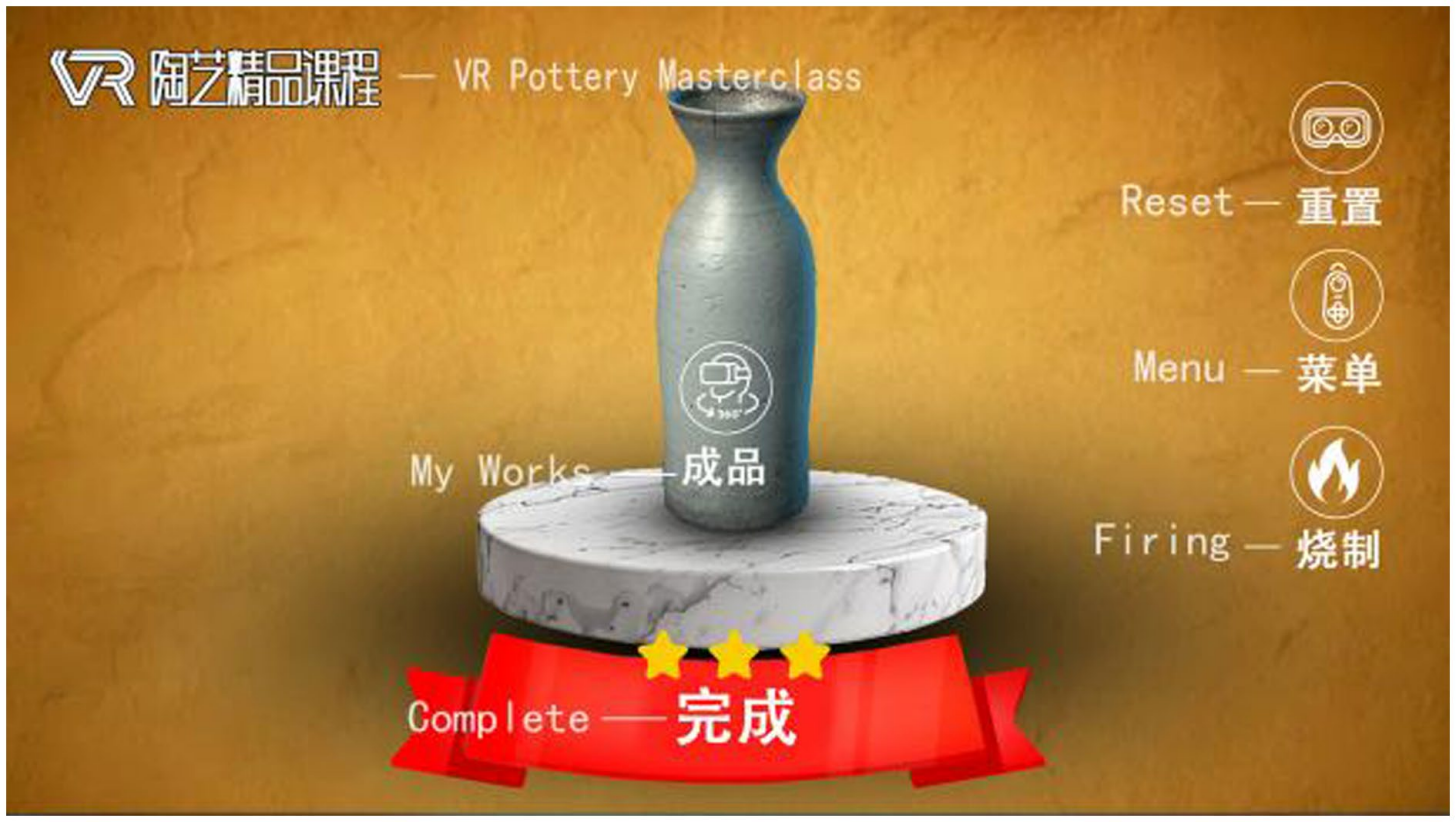
Completion interface displaying the finished pottery work, achievement indicators, and system feedback.

This study used an all-in-one VR headset produced by Pico, which is known for its affordability and user-friendly interface, making it well-suited for children and home use. The virtual pottery system was designed based on cognitive engagement and affective arousal theories, integrating multimodal interactive features. In the virtual pottery course, students first perform basic clay shaping in a simulated 3D environment. They then engage in detailed carving and decoration using specific gestures and virtual tools. Finally, they place the completed piece into a virtual kiln for firing, where they can observe the result. To enhance usability, the system incorporates dynamic highlights to indicate actionable areas, spatial projection arrows to show the workflow direction, and progressive task decomposition to reduce operational complexity. During the firing stage, particle-based flame effects and rousing sound effects are used to create an immersive atmosphere. Upon completion, the final work is presented through a 360° rotating display accompanied by achievement badges, which enhance the learner’s sense of accomplishment. Figures 2–5 depict this process.

### 2.2 Research design and sampling

Quantitative data were collected using a structured questionnaire. The questionnaire was designed based on the dual-path model of cognitive engagement and affective arousal, incorporating localized adaptations of eight well-established constructs (Table 1). A seven-point Likert scale was used (1 = strongly disagree, 7 = strongly agree), with positively and negatively worded items (e.g., CL1 and CL2) to control for response bias. In total, two professional translation scholars were invited to perform a bidirectional translation of the questionnaire to ensure cross-cultural validity and linguistic accuracy. First, the original English questionnaire was translated into Chinese to ensure that parents of the child participants could clearly understand the items. The Chinese questionnaire was then back-translated into English to verify semantic consistency with the original scale.

**Table 1:** Measurement items for the dual-path model of cognitive engagement and emotional arousal.

Construct	Item code	Item content	Source
Attention guidance	AG1	Visual highlighting in the system directs my attention to key steps.	Parong and Mayer (2018)
	AG2	Dynamic arrows help me accurately predict the next operation step.	
	AG3	Color-changing signals timely remind me to correct mistakes.	
Cognitive load	CL1	The mental effort required to complete tasks feels overwhelming.	Kalyuga (2011)
	CL2	I struggle to concentrate when handling multiple steps at once.	
	CL3	Task complexity is well matched with my skill level.	
Valence design	VD1	The realistic textures of virtual materials bring me pleasant tactile associations.	Plass et al. (2020)
	VD2	Particle effects during firing enhance my anticipation of the final result.	
	VD3	The artistic design of tools stimulates my creativity.	
Arousal design	AD1	The tactile feedback during shaping makes my heart beat faster.	Um et al. (2012)
	AD2	The countdown sound during firing increases my breathing rate.	
	AD3	Vibrations triggered by errors make my muscles tense.	
Flow	FL1	I become fully immersed in creation and lose track of time.	Jackson and Marsh (1996)
	FL2	I can clearly perceive progress through each operation step.	
	FL3	The task challenge is well balanced with my skill level.	
Satisfaction	SA1	The 3D display of my finished work gives me a sense of achievement.	Makransky and Lilleholt (2018)
	SA2	Unlocking achievement badges motivates me to keep learning.	
	SA3	Sharing my work with others makes me happy.	
Behavioral intention	BI1	I plan to continue learning new skills through this VR pottery course.	Venkatesh et al. (2012)
	BI2	I would recommend this course to friends to improve their pottery skills.	
	BI3	I would practice this course content voluntarily, even without homework.	
	BI4	I would try new features as soon as the course updates.	
Perceived learning achievement	LA1	I can independently complete all the basic shapes taught in the course.	Loksa et al. (2016)
	LA2	I’ve mastered the technique of controlling clay thickness.	
	LA3	I can accurately predict the glaze color outcome based on the formula.	
	LA4	I can apply course techniques to create original pottery designs.	

All items were rated by parents based on observation and discussion with their child immediately after the virtual reality session, reflecting parent-reported perceptions of the child’s internal states (e.g., arousal, cognitive load, and flow). AG, attention guidance; CL, cognitive load; VD, valence design; AD, arousal design; FL, flow; SA, satisfaction; BI, behavioral intention; LA, perceived learning achievement.

The questionnaire was designed to measure eight constructs based on the dual-path model of cognitive engagement and affective arousal (Table 1). All items were completed by parents based on observation and discussion with their child immediately after the VR session, thereby capturing parent-reported perceptions of the child’s internal states.

A pre-test was conducted with 30 participants to assess system functionality and survey clarity. It focused on (1) technical barriers in the VR pottery system workflow, (2) accurate comprehension of and ability to follow task instructions, (3) stability of VR system interaction, (4) ambiguities in the questionnaire items, (5) children’s comprehension of the Likert scale, and (6) consistency of response logic in reverse-coded items. The pilot test results showed that the system had a functional availability rate of 96.7%, and the overall reliability coefficient of the questionnaire was 0.91 (Cronbach’s *α* > 0.9). The reverse-coded items were significantly negatively correlated with the total score, and children demonstrated an accuracy rate of more than 92% in understanding the scale items.

### 2.3 Participants and data collection

This study was conducted in four major Chinese cities: Beijing, Shanghai, Guangzhou, and Shenzhen. A total of 220 participants were recruited from eight local pottery training institutions. All participants were elementary school students aged 7–12 years. This age range spans important developmental transitions in attention control, motor coordination, and emotional regulation, which may influence how children experience flow, satisfaction, and cognitive load. Each participant had prior experience completing a pottery project. This criterion was implemented to ensure that all children had a baseline understanding of tactile pottery processes, thereby controlling for the confounding effect of the total novelty of the task itself. Each participant underwent a 10-min VR pottery session, which included clay shaping, detail carving, and virtual firing.

**Figure 6:**
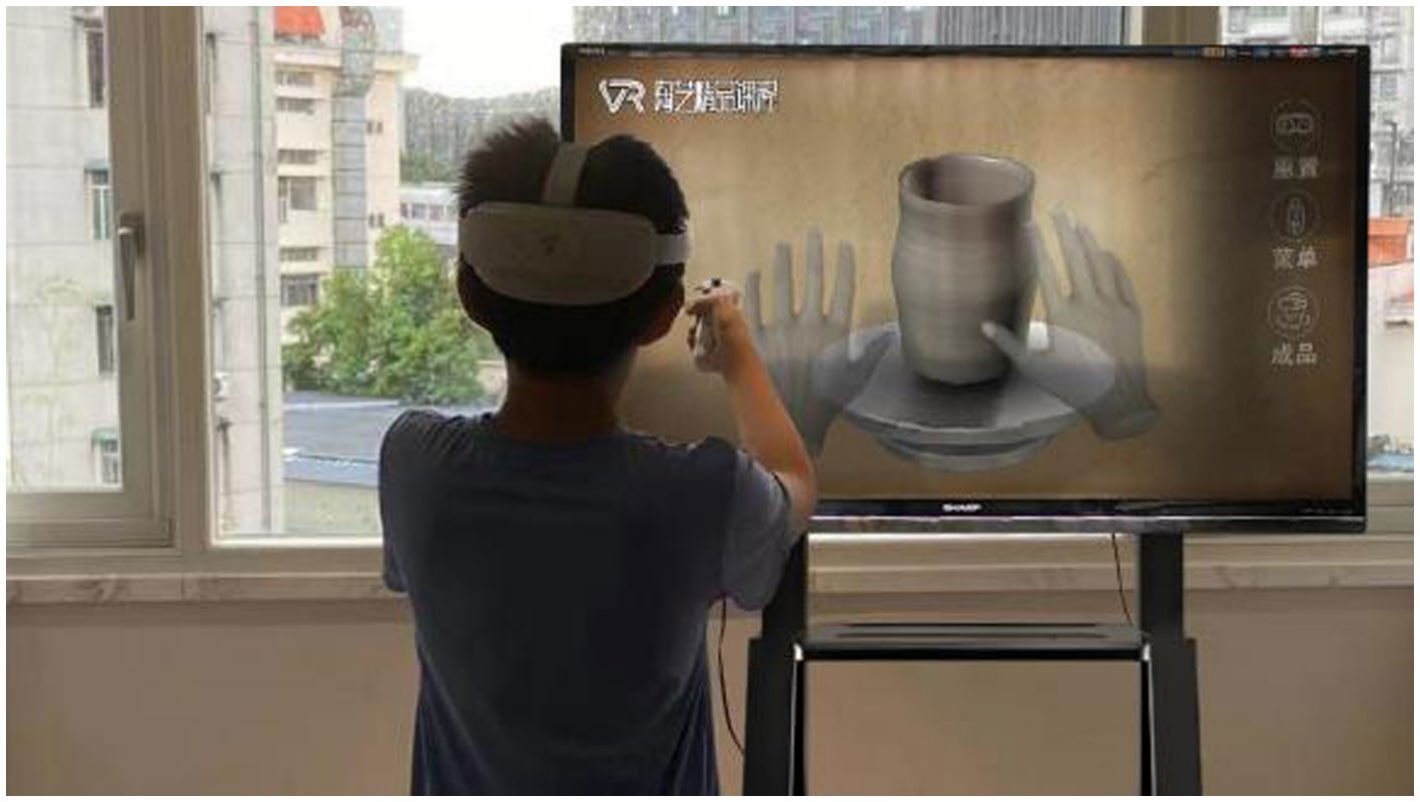
Child participant using the VR pottery system during the experimental session.

**Figure 7:**
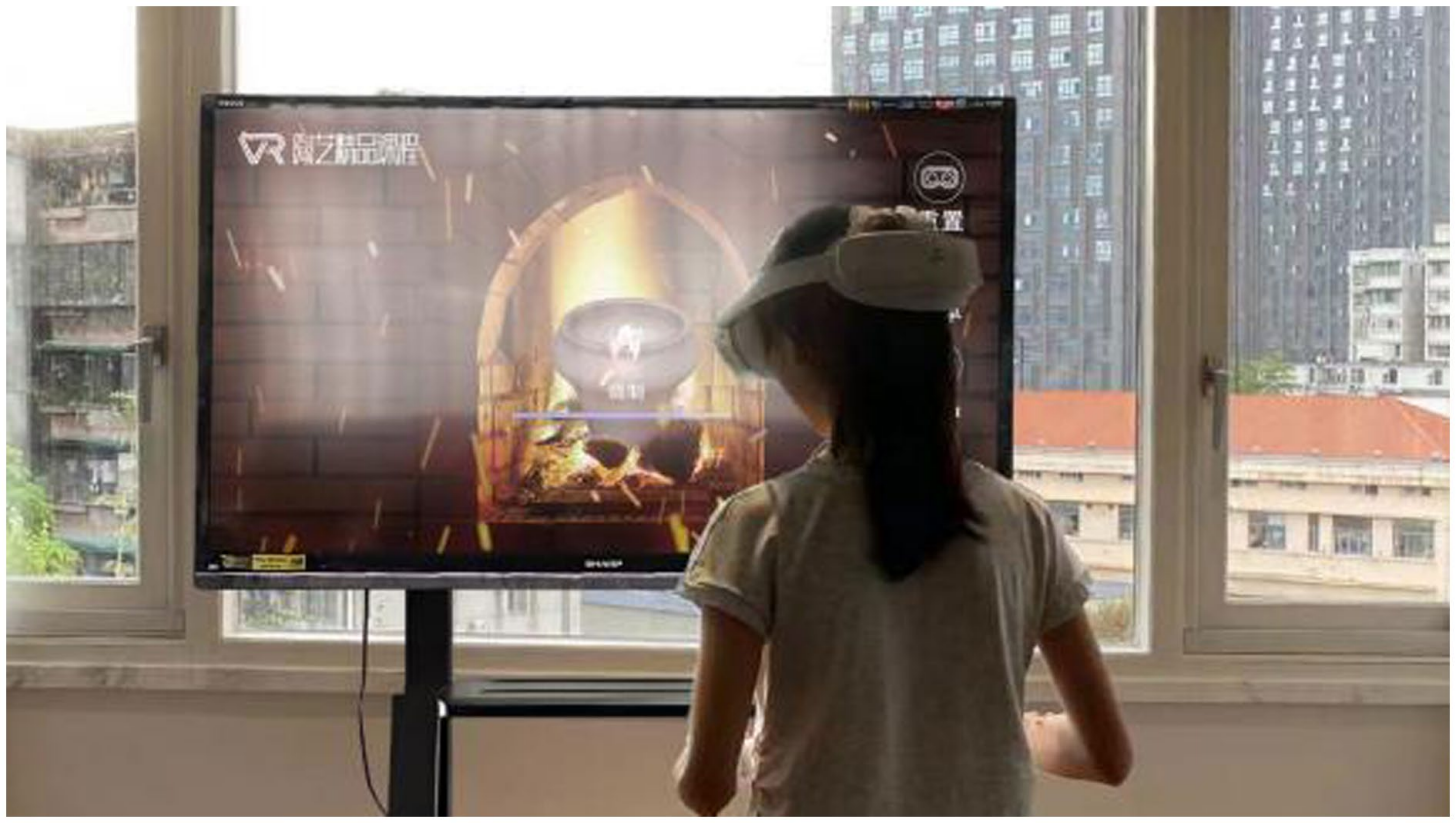
Experimental environment showing a participant interacting with the VR pottery system in front of a display monitor.

The questionnaire was designed using the professional version of “Wenjuanxing” (a Chinese online survey platform), printed, and manually completed on-site by the parents. Questionnaires completed in less than 3 min or those with inconsistent responses to reverse-coded items were excluded, resulting in 207 valid responses. Figures 6, 7 show scenes from the VR pottery sessions.

Parents manually completed the questionnaire on-site based on their observation of and communication with their child immediately after the VR experience. Although this method was pragmatic for a young sample and ensured a high response rate, it yielded an indirect measure of the child’s internal state and may be subject to parental interpretation bias.

Although self-report is generally preferred for assessing internal psychological states, the developmental characteristics of children aged 7–12 years pose challenges for reliable self-completion of Likert scale questionnaires (Borgers et al., 2000). Therefore, this study adopted a parent-proxy report approach, a method that has been widely employed in research on children’s experiences with technology and media (Livingstone and Helsper, 2008; Rideout, 2017). Parents were instructed to discuss each questionnaire item with their child immediately after the VR session and to record the child’s response, thereby integrating observation with the child’s verbal report to approximate the child’s subjective perspective. Consequently, all measures of the children’s internal states (e.g., attention guidance, cognitive load, valence design, arousal design, flow, satisfaction, behavioral intention, and perceived learning achievement) reflected parent-reported perceptions of the child’s experience. Although this method may introduce some degree of interpretation bias, it represents a pragmatic and commonly accepted strategy in developmental and educational research when direct child self-report is impractical or unreliable (Achenbach et al., 1987).

Furthermore, a substantial portion (55.1%) of our participants had no prior VR experience in the past 3 months, making novelty a potential confounding factor that could inflate reports of flow and satisfaction. We attempted to mitigate this by including a familiarization phase before the formal task.

The entire experiment was conducted under parental supervision, and the guardians signed a double-blind consent form that complied with the Ethical Review Measures for Biomedical Research Involving Humans. This study was reviewed and approved by the Ethics Committee of the School of Fine Arts and Design, Guangzhou University (Approval Code: 2025009).

### 2.4 Statistical analysis

The data were analyzed using SPSS 27.0 and SmartPLS 4.0. SmartPLS was deemed suitable for this research because it handles small sample sizes efficiently, does not require a strict multivariate normal distribution, and is particularly effective for exploratory model testing, making it highly compatible with the study’s aim.

First, the reliability of the eight questionnaire constructs was assessed using SPSS with Cronbach’s *α* coefficients to evaluate internal consistency. Subsequently, SmartPLS was used to assess the quality of the measurement model by calculating the average variance extracted (AVE) and composite reliability to verify convergent validity. The Fornell–Larcker criterion and the heterotrait–monotrait (HTMT) ratio were used to examine discriminant validity. A correlation matrix of the eight latent variables was constructed to ensure that each variable’s self-explanatory power exceeded its correlation with other constructs, and HTMT thresholds were used to mitigate redundancy risks. Subsequently, a structural model analysis was conducted using SmartPLS, employing bootstrapping with 5,000 resamples to test the significance of the path coefficients.

T-values were used to evaluate the statistical strength of deviation from the null hypothesis, and *p*-values were used to quantify the probability of relationships arising due to random error, precisely estimating the direction and strength of the relationships between variables. The *f*^2^ effect sizes were also computed to evaluate the differential impact of cognitive and emotional dimensions on flow and satisfaction, as well as the respective effects of flow and satisfaction on behavioral intention and perceived learning achievement. The final model integrated the causal chain of the eight variables, revealing the dual-path mechanism of cognitive engagement and emotional arousal in children’s VR-based pottery learning and its underlying logic in driving sustained usage intention.

## 3 Results

### 3.1 Demographic characteristics

Table 2 presents the participants’ demographic characteristics. The 207 children showed a relatively balanced sex distribution: 53.6% male and 46.4% female. The majority were aged 9–10 years (45.0%), followed by those aged 7–8 years (30.9%) and 11–12 years (24.1%). Regarding familiarity with digital devices, 61.4% of the participants could independently perform basic operations, 27.0% demonstrated advanced digital proficiency, and 11.6% were unfamiliar and required parental assistance.

**Table 2:** Demographic profile of the participants.

Characteristic	Option	Frequency	Percentage (%)
Sex	Male	111	53.6%
	Female	96	46.4%
Age group (years)	7–8	64	30.9%
	9–10	93	45.0%
	11–12	50	24.1%
Familiarity with digital devices	Completely unfamiliar (requires parental assistance)	24	11.6%
	Can independently complete basic tasks (e.g., tap and swipe)	127	61.4%
	Proficient (e.g., downloading apps and playing games)	56	27.0%
Parents’ highest educational level	High school or below	41	19.8%
	Junior college	62	30.0%
	Bachelor’s degree	72	34.8%
	Master’s degree	21	10.1%
	Doctorate	11	5.3%
Annual household income (CNY 1,000)	0–100	31	15.0%
	100–200	62	30.0%
	200–350	72	34.8%
	350–500	31	15.0%
	> 500	11	5.3%
Any family member in arts/education-related profession	Yes	72	34.8%
	No	135	65.2%
Parental attitude toward children using VR	Strongly supportive	83	40.1%
	Moderately supportive	93	44.9%
	Neutral	21	10.1%
	Concerned about potential risks	10	4.8%
Frequency of VR use in the past three months	Never	114	55.1%
	1–3 times	62	30.0%
	Once per month	21	10.1%
	Once or more per week	10	4.8%
Does the household own a VR device?	Yes	41	19.8%
	No, requires renting or borrowing	114	55.1%
	Depends on school or institutional access	52	25.1%

VR, virtual reality. Income is reported in thousands of Chinese Yuan (CNY).

Parents’ education levels were generally moderate to high, with 34.8% holding a bachelor’s degree, 30.0% holding an associate degree, and 15.4% holding a graduate degree or above. Regarding annual household income, 64.8% of families reported earnings between CNY¥100,000 and CNY¥350,000, with 34.8% specifically earning between CNY¥200,000 and CNY¥350,000. All income figures are reported in Chinese Yuan (RMB). Approximately 34.8% of households had family members working in arts- or education-related professions.

Parental attitudes toward children’s use of VR technology were largely positive: More than 85.0% expressed full or moderate support, with 40.1% reporting being “highly supportive.” Regarding recent usage, 55.1% of the children had not used VR devices in the past 3 months, and only 4.8% used them weekly or more frequently. In terms of device ownership, 19.8% of households owned a VR device, while 55.1% relied on rentals or borrowing and 25.1% accessed VR through schools or other institutions.

### 3.2 Measurement model

The measurement model demonstrated good reliability (Table 3) and convergent and discriminant validity. All constructs had Cronbach’s *α* coefficients above the acceptable threshold of 0.7, indicating strong internal consistency reliability. In addition, composite reliability values exceeded the recommended threshold of 0.7, and AVE values were above the minimum standard of 0.5, confirming the robustness of convergent validity. Although the AVE of behavioral intention was slightly below 0.7, it significantly exceeded the critical threshold of 0.5 and, therefore, was considered acceptable.

**Table 3:** Reliability and validity tests for the measurement model.

Factor	Items	Loadings	Cronbach’s alpha	rho_A	CR	AVE
AG	AG1	0.866	0.816	0.818	0.89	0.73
	AG2	0.849				
	AG3	0.848				
CL	CL1	0.871	0.829	0.84	0.898	0.745
	CL2	0.829				
	CL3	0.888				
VD	VD1	0.86	0.794	0.801	0.879	0.708
	VD2	0.802				
	VD3	0.861				
AD	AD1	0.905	0.854	0.866	0.911	0.774
	AD2	0.854				
	AD3	0.879				
FL	FL1	0.873	0.897	0.899	0.928	0.763
	FL2	0.894				
	FL3	0.846				
	FL4	0.881				
SAT	SAT1	0.857	0.866	0.868	0.918	0.789
	SAT2	0.912				
	SAT3	0.895				
BI	BI1	0.88	0.841	0.854	0.893	0.677
	BI2	0.815				
	BI3	0.802				
	BI4	0.791				
LA	LA1	0.906	0.872	0.883	0.913	0.724
	LA2	0.814				
	LA3	0.823				
	LA4	0.857				

AVE, average variance extracted; CR, composite reliability; AG, attention guidance; CL, cognitive load; VD, valence design; AD, arousal design; FL, flow; SAT, satisfaction; BI, behavioral intention; LA, perceived learning achievement.

The square root of the AVE for each construct was greater than its correlation with any other construct, supporting discriminant validity according to the Fornell–Larcker criterion (Table 4). Furthermore, all HTMT values were below the conservative threshold of 0.85, confirming that each construct was sufficiently distinct from the others (Table 5). These results indicate that the measurement model possesses sound psychometric properties, providing a reliable foundation for subsequent structural analyses.

**Table 4:** Discriminant validity (Fornell–Larcker criterion).

	Attention guidance	Cognitive load	Valence design	Arousal design	Flow	Satisfaction	Behavioral intention	Perceived learning achievement
A-AG	0.854							
B-CL	0.441	0.863						
C-VD	0.34	0.407	0.841					
D-AD	0.204	0.404	0.21	0.88				
FL	0.471	0.446	0.304	0.361	0.874			
SAT	0.445	0.465	0.373	0.258	0.321	0.888		
Y-BI	0.459	0.324	0.369	0.227	0.62	0.485	0.823	
Z-LA	0.334	0.266	0.162	0.154	0.367	0.278	0.369	0.851

AG, attention guidance; CL, cognitive load; VD, valence design; AD, arousal design; FL, flow; SAT, satisfaction; BI, behavioral intention; LA, perceived learning achievement.

**Table 5:** HTMT ratio matrix of latent constructs.

	Attention guidance	Cognitive load	Valence design	Arousal design	Flow	Satisfaction	Behavioral intention	Perceived learning achievement
A-AG								
B-CL	0.531							
C-VD	0.416	0.499						
D-AD	0.248	0.474	0.246					
F	0.55	0.511	0.353	0.413				
SAT	0.525	0.548	0.451	0.295	0.363			
Y-BI	0.55	0.38	0.447	0.264	0.706	0.562		
Z-LA	0.39	0.309	0.192	0.174	0.41	0.316	0.423	

HTMT, heterotrait–monotrait; AG, attention guidance; CL, cognitive load; VD, valence design; AD, arousal design; FL, flow; SAT, satisfaction; BI, behavioral intention; LA, perceived learning achievement.

### 3.3 Structural model

The structural model analysis results (Table 6) confirmed the hypothesized path relationships. A majority of the hypothesized relationships were statistically significant (*p* < 0.05), thereby supporting the proposed hypotheses. Specifically, attention guidance had a significant positive effect on flow (*β* = 0.319, *p* < 0.001) and satisfaction experiences (*β* = 0.263, *p* < 0.001), supporting H1a and H1b. Cognitive load control also significantly influenced both flow (*β* = 0.194, *p* = 0.023) and satisfaction (*β* = 0.255, *p* = 0.002), confirming H2a and H2b. Valence design had a significant positive effect on satisfaction (*β* = 0.165, *p* = 0.016), supporting H3a. However, it did not have a significant effect on flow experience (*β* = 0.074, *p* = 0.307); therefore, H3b was not supported. Arousal design significantly affected flow experience (*β* = 0.202, *p* = 0.005), confirming H4a; however, its effect on satisfaction (*β* = 0.067, *p* = 0.396) was not supported; therefore, H4b was not supported. Flow experience had a strong, significant positive impact on behavioral intention (*β* = 0.518, *p* < 0.001), thereby supporting H5. Satisfaction experience also significantly influenced behavioral intention (*β* = 0.319, *p* < 0.001), confirming H6. Finally, behavioral intention significantly predicted perceived learning achievement (*β* = 0.369, *p* < 0.001), supporting H7.

**Table 6:** Path coefficients and significance testing results.

Path	Original sample (O)	Sample mean (M)	Standard deviation (STDEV)	*T* value	*p*-value	95% CI lower (2.50%)	95% CI upper (97.50%)	Conclusion
A-AG → FL	0.319	0.321	0.072	4.423	0.000	0.173	0.453	Hypothesis supported
A-AG → SAT	0.263	0.265	0.066	3.984	0.000	0.132	0.39	Hypothesis supported
B-CL → FL	0.194	0.197	0.086	2.267	0.023	0.03	0.362	Hypothesis supported
B-CL → SAT	0.255	0.243	0.08	3.171	0.002	0.077	0.394	Hypothesis supported
C-VD → FL	0.074	0.074	0.072	1.021	0.307	−0.072	0.209	Hypothesis not supported
C-VD → SAT	0.165	0.167	0.068	2.416	0.016	0.031	0.3	Hypothesis supported
D-AD → FL	0.202	0.196	0.073	2.779	0.005	0.051	0.331	Hypothesis supported
D-AD → SAT	0.067	0.07	0.079	0.848	0.396	−0.081	0.225	Hypothesis not supported
FL → Y-BI	0.518	0.52	0.049	10.568	0.000	0.417	0.612	Hypothesis supported
SAT → Y-BI	0.319	0.32	0.055	5.776	0.000	0.207	0.424	Hypothesis supported
Y-BI → Z-LA	0.369	0.378	0.076	4.841	0.000	0.222	0.519	Hypothesis supported

CI, confidence interval; AG, attention guidance; CL, cognitive load; VD, valence design; AD, arousal design; FL, flow; SAT, satisfaction; BI, behavioral intention; LA, perceived learning achievement. All constructs in this table were assessed via parent reports (see Section 2.3).

**Figure 8:**
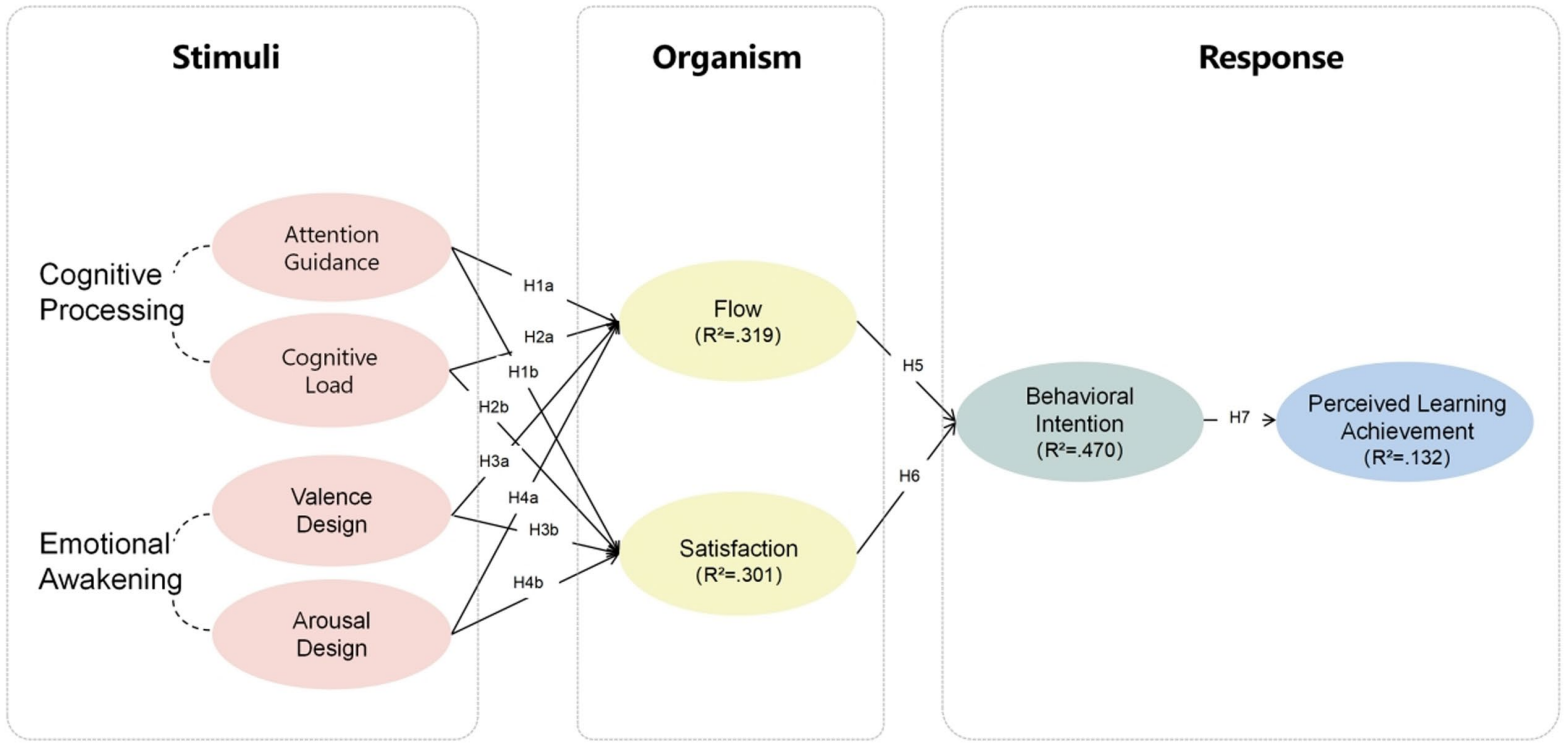
Structural model results of the proposed S–O–R framework with standardized path coefficients and explained variances.

In terms of explanatory power (Figure 8), the antecedents (AG, CL, VD, and AD) explained 31.9 and 30.1% of the variance in flow and satisfaction, respectively. Flow and satisfaction jointly explained 47.0% of the variance in behavioral intention, indicating a moderate to substantial level of explanatory power. Behavioral intention explained 13.2% of the variance in perceived learning achievement.

## 4 Discussion

### 4.1 General discussion

This study explored the dual-path collaborative mechanism of cognition and emotion. The two cognitive engagement variables—attention guidance and cognitive load control—had significant positive effects on children’s flow and satisfaction in the VR-based pottery course. Specifically, attention guidance positively affected flow and satisfaction, indicating that children are highly sensitive to interface cues, such as process guidance and color highlights. This finding aligns with those reported by Parong and Mayer (2018), who noted that “cues enhance immersive experience.” Furthermore, cognitive load control significantly influenced flow and satisfaction, reflecting the effectiveness of Sweller’s (2011) cognitive load theory, which suggests that task difficulty that matches children’s cognitive capacity significantly enhances their sense of control and accomplishment. These two paths validate the benefits of “cognitive-assistive design” in VR-based education and emphasize the importance of reducing cognitive cost and enhancing interface comprehensibility for child users (Sulisworo et al., 2024).

In the emotional path, valence design had a limited impact on flow but a significant effect on satisfaction, supporting the findings of Guerra-Tamez (2023), who concluded that “immersive visuals significantly enhance flow experience.” This suggests that aesthetic elements, such as high-fidelity textures, color gradients, and particle effects, can significantly strengthen children’s emotional resonance and enjoyment. In contrast, arousal design had a stronger influence on flow but a weaker and non-significant effect on satisfaction. This finding suggests that tactile and auditory stimuli effectively enhance emotional engagement and arousal but may not increase satisfaction. This aligns with the findings of Um et al. (2012), who argued that “arousal should be moderated in intensity,” suggesting that designers should avoid overstimulation to avoid disrupting immersion.

Finally, the study confirmed that both flow and satisfaction significantly predict children’s continued usage intention. Specifically, the paths flow → intention and satisfaction → intention closely align with the findings of Chiu and Chen (2023) in distance learning, highlighting that emotional and immersive learning experiences significantly enhance continued participation willingness. Furthermore, continued usage intention had a significant effect on perceived learning achievement, suggesting that frequent practice can improve skills and enhance creativity (Wang et al., 2024).

Further analysis confirmed the dominance of cognitive engagement over emotional arousal. The combined effect of attention guidance and cognitive load control on flow (*β* = 0.513) substantially exceeded that of valence and arousal design (*β* = 0.276). Similarly, the total effect of cognitive engagement on satisfaction (*β* = 0.518) outweighed that of emotional arousal (*β* = 0.235). This finding indicates that mechanisms such as rational judgment and task fit are more decisive than sensory stimulation in shaping children’s immersive states and overall experience evaluation. These findings align with those of meta-analyses, indicating that optimizing learning tasks by reducing extraneous cognitive load is crucial for enhancing focus and motivation in VR instruction (Makransky and Petersen, 2019). For beginners or child users, emotional arousal design lacking clear cognitive guidance may lead to distractions (Makransky and Petersen, 2019). Therefore, in child-centered immersive design, cognitive engagement structures should be prioritized, whereas emotional arousal should serve as an auxiliary enhancement.

It is important to acknowledge that the brief 10-min exposure and the high proportion of first-time VR users (55.1%) may have induced a novelty effect, potentially inflating the reported levels of flow, satisfaction, and continued usage intention. Consequently, our findings primarily reflect children’s immediate responses to a novel immersive experience rather than their long-term engagement patterns. Future longitudinal studies are needed to examine whether these intentions translate into sustained usage over time and to disentangle the novelty effect from genuine learning motivation.

### 4.2 Theoretical implications

This study developed a structural model based on the S–O–R framework to investigate children’s continued usage intention in VR-based pottery courses. It systematically explored how two categories of external stimuli—cognitive engagement and emotional arousal—influence behavioral intention and perceived learning achievement through the mediating effects of flow and satisfaction experiences. This forms a compound path of “cognitive-emotional resonance and psychological-behavioral linkage.” This integrative perspective is rare in current VR-based education research and offers three key theoretical contributions. First, it develops a compound motivational model grounded in the S–O–R framework, thereby expanding the theoretical scope of behavioral intention research in VR-based education. Second, it introduces a dual-variable mechanism of flow and satisfaction at the experiential level, deepening our understanding of situational experiences in immersive education. Third, it emphasizes the dominant role of cognitive engagement in younger users, enriching and refining theories on age-appropriate immersive learning for children.

Overall, the “cognitive-emotional dual-path collaborative mechanism” proposed based on the S–O–R framework was validated in this study. It clarified the independent effects of cognitive engagement (e.g., attention guidance and cognitive load control) and emotional arousal (e.g., valence and arousal design) on psychological experiences and demonstrated their potential amplifying interactions. Furthermore, this study extended the application of the S–O–R model to the context of art education and child users. The S–O–R model has been widely applied in areas such as digital advertising, virtual exhibitions, and adult vocational training. However, this study extended it to children’s VR-based art education, providing preliminary evidence of its potential explanatory value for immersive learning experiences. This is particularly relevant for users aged 7–12 years, who tend to show heightened sensitivity to stimulus-driven psychological responses. Overall, although the S–O–R framework helped structure the relationships examined in this study, the evidence should be interpreted as preliminary rather than conclusive. Although the findings suggest the potential explanatory value of the S–O–R model for understanding children’s immersive learning experiences, given this study’s cross-sectional design, subjective self-report measures, and limited sample representativeness, future longitudinal and multi-method research is needed for a more robust validation of these pathways.

### 4.3 Policy implications

Considering China’s push for educational digital transformation and the widespread adoption of immersive technologies, the General Office of the State Council released the Action Plan for Promoting the Modernization of Education through Digitalization in 2024. This plan clearly sets the goal of establishing a national intelligent education platform covering 100 million teachers and students by 2027 and includes the deployment of 10,000 immersive classroom demonstration sites under the “Digital Campus Construction Project.” Therefore, this study offers preliminary insights with policy implications for developing digital art education in primary schools.

First, this study provides quantifiable reference dimensions for curriculum design standards in children’s digital art education. By structurally categorizing cognitive engagement and emotional arousal, it identifies “attention guidance,” “cognitive load control,” and “aesthetic emotional arousal” as key factors influencing children’s immersive experience and behavioral intention. These variables can be translated into evaluation indicators of course design quality, serving both digital course assessment and government selection processes. Second, the study highlights disparities between children’s operational skills and family background, emphasizing that emotional disturbances and sensory overload can significantly affect children’s experiences in VR environments. These insights do not constitute direct policy recommendations but instead offer preliminary considerations that may inform future discussions on integrating VR into children’s art education.

### 4.4 Practical implications

This study provides concrete practical references and actionable guidance for the content design and system development of VR-based pottery courses. Building on the finding that cognitive engagement is the primary driver of flow and satisfaction, the following design priorities are proposed.

First, for content development, the cognitive and emotional dual-path variable framework can provide a functional template. Developers should embed features such as “process guidance cues” and “task grading mechanisms” to establish a solid cognitive foundation. Subsequently, “immersive audio feedback” and “achievement-based visual effects” can be integrated to enhance emotional engagement.

Second, this study provides a clear priority structure for system development teams. Attentional guidance should be considered the primary factor in directing the learning process. Therefore, developers should prioritize the rationality and continuity of task workflows before incorporating secondary emotional elements, such as visual aesthetics and animation effects. Regarding hardware compatibility, appropriately matching haptic feedback with visual clarity is crucial to avoid excessive sensory stimulation, which could undermine children’s operational confidence and primary cognitive experience.

Importantly, our findings underscore that, for children, sensory stimulation alone (e.g., vivid effects or arousing sounds) is insufficient to generate deep satisfaction without being anchored by clear cognitive guidance. Designers should therefore ensure that affective elements are always integrated with, rather than substituted for, well-structured cognitive support.

### 4.5 Limitations and future research

Although this study obtained meaningful results in both theoretical modeling and empirical validation, it has several limitations that call for refinement and extension in future research. First, the study has limitations regarding the participant sample. The sample was drawn exclusively from major metropolises in China, which may limit the generalizability of the findings. Children from rural areas or with different socioeconomic, educational, and cultural backgrounds may differ in how they interact with VR learning.

Second, the age range of 7–12 years encompasses significant developmental diversity in cognitive, motor, and emotional capacities. Although our model provides an overall picture, it may mask age-specific engagement patterns. Furthermore, requiring participants to have prior pottery experience was necessary to control for task novelty; however, it may have introduced a “familiarity bias,” as these children likely compared their VR experience to their tactile memories of real clay.

Third, the research design and measurement methods present several constraints. The cross-sectional survey design revealed potential path mechanisms among variables but could not establish temporal or causal order. In particular, the relationship between “continued usage intention” and “perceived learning achievement” requires longitudinal tracking to verify whether behavioral intentions translate into sustained capability development.

Fourth, our measurement approach relied on parent-proxy reports rather than children’s direct self-reports. Although this method is commonly employed in research with young children (Achenbach et al., 1987; Borgers et al., 2000) and was implemented through structured parent–child discussions immediately after the VR session, it may still have introduced interpretation bias. Parents’ observations and their children’s verbal expressions may not fully capture the children’s internal states, such as flow, cognitive load, or affective arousal, with complete accuracy. This reliance on proxy reporting represents an inherent limitation of the current study.

Fifth, a substantial proportion of the participants were first-time VR users, and the potential “novelty effect” may have transiently elevated self-reported engagement and satisfaction metrics. Moreover, the measurement of perceived learning achievement relied on self-reported perceptions rather than objective assessments. Although suitable for capturing children’s self-perceived competence, this approach may not fully reflect actual skill acquisition and limits the objective validation of skill transfer.

Finally, cultural differences may have influenced the participants’ experiences with and perceptions of the VR-based pottery course.

Future research should pursue the following directions to enrich and deepen the theoretical and practical understanding of VR-based pottery education. First, researchers could diversify the sample structure to include a wider range of participant groups, such as children from diverse geographic and socioeconomic backgrounds, adolescents, and adult users, for comparative analysis. Extending this to include children with no prior pottery experience would further clarify how prior material knowledge shapes engagement in digital simulations. In addition, conducting age-based subgroup analyses with larger samples, such as comparing children aged 7–8, 9–10, and 11–12 years, would reveal developmental trajectories in cognitive engagement, emotional response, and behavioral persistence, thereby offering a more comprehensive and developmentally nuanced understanding of VR learning mechanisms.

Second, to address the limitations of parent-proxy reporting, future studies should employ child-appropriate self-report instruments that enable more direct assessment of children’s internal states. These may include visual analog scales, smiley face Likert scales, or pictorial measures specifically designed for young children (Read and MacFarlane, 2006). Researchers could also incorporate real-time physiological measures (e.g., heart rate variability and skin conductance) as complementary indicators of affective arousal and cognitive load, thereby triangulating subjective reports with objective data.

Third, longitudinal, periodic tracking and experimental interventions should be incorporated to verify causal pathways and observe dynamic changes in children’s flow experience, learning motivation, and skill acquisition as they engage in the VR-based pottery course. This would clarify whether psychological experiences can truly translate into long-term behavioral and learning outcomes.

Fourth, VR-based pottery systems should be continuously refined and promoted. The research team plans to enhance the user experience of the VR-based pottery course and expand its implementation to broader educational contexts.

Fifth, perceived learning achievement was measured based on self-reported perceptions rather than objective assessments. Although this approach is suitable for an exploratory study that captures children’s self-perceived competence, it may not fully reflect actual skill acquisition. Future research could incorporate objective measures, such as expert evaluations of the creativity or technical quality of the final pottery pieces, task completion accuracy, or pre–post skill tests, to validate the link between continued usage intention and tangible learning outcomes.

## Data Availability

The original contributions presented in the study are included in the article/supplementary material, further inquiries can be directed to the corresponding author.
